# Supplementary figures and images for: Computational design and interpretation of single-RNA translation experiments
Source: PLoS Comput Biol. 2019 Oct 16;15(10):e1007425. doi: 10.1371/journal.pcbi.1007425 (PMC6816579; doi:10.1371/journal.pcbi.1007425)

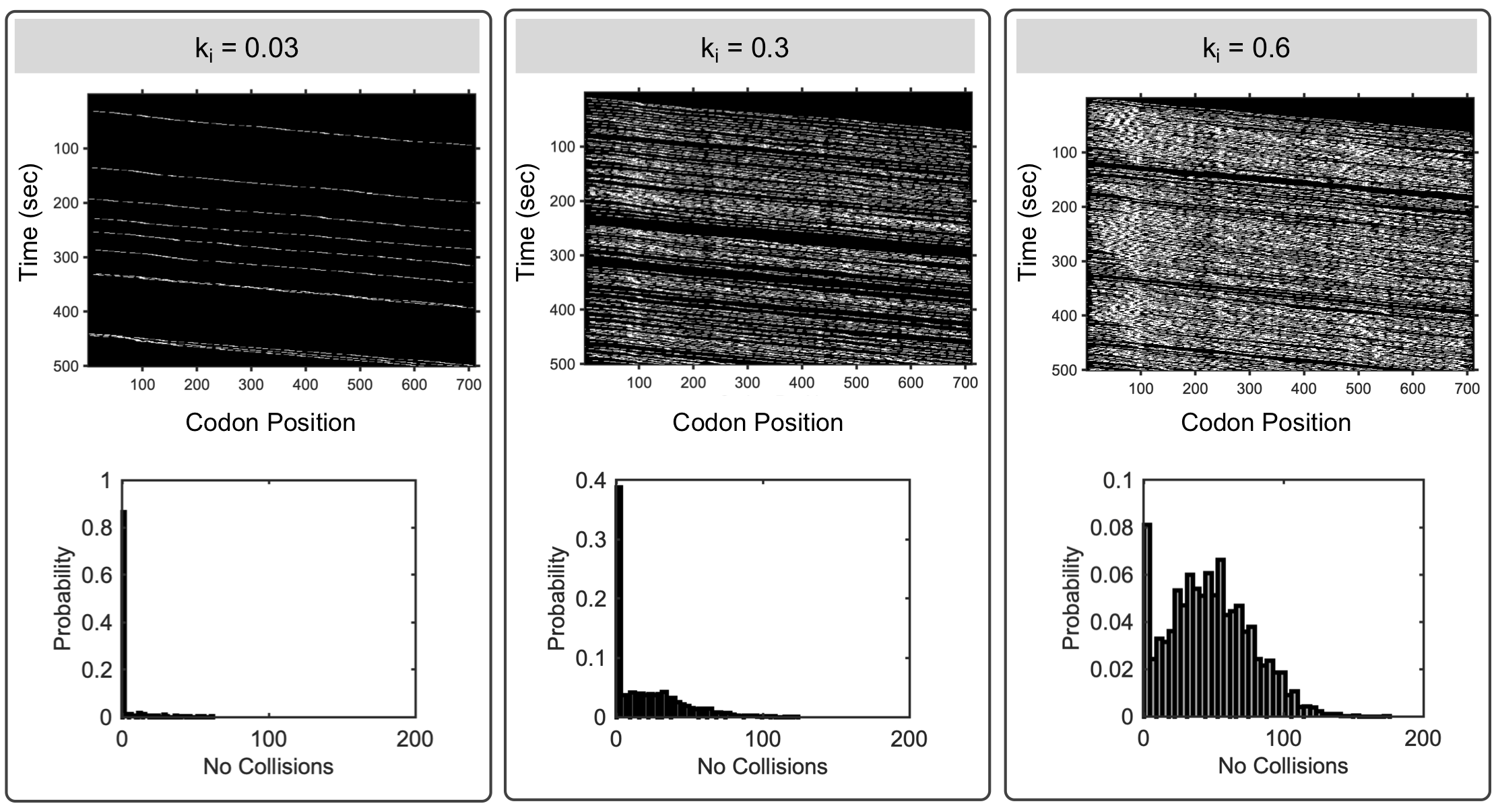

Supplement: S1 Fig — Translation was simulated using the β-actin gene, varying initiations rates from 0.03 to 0.6, a constant elongation (ke = 10 aa/sec), and a ribosomal footprint of 9 codons. Top panels show a kymograph of the ribosome movement. Lower panels show the distribution of collisions for each ki. (TIF) [file pcbi.1007425.s001.tif]

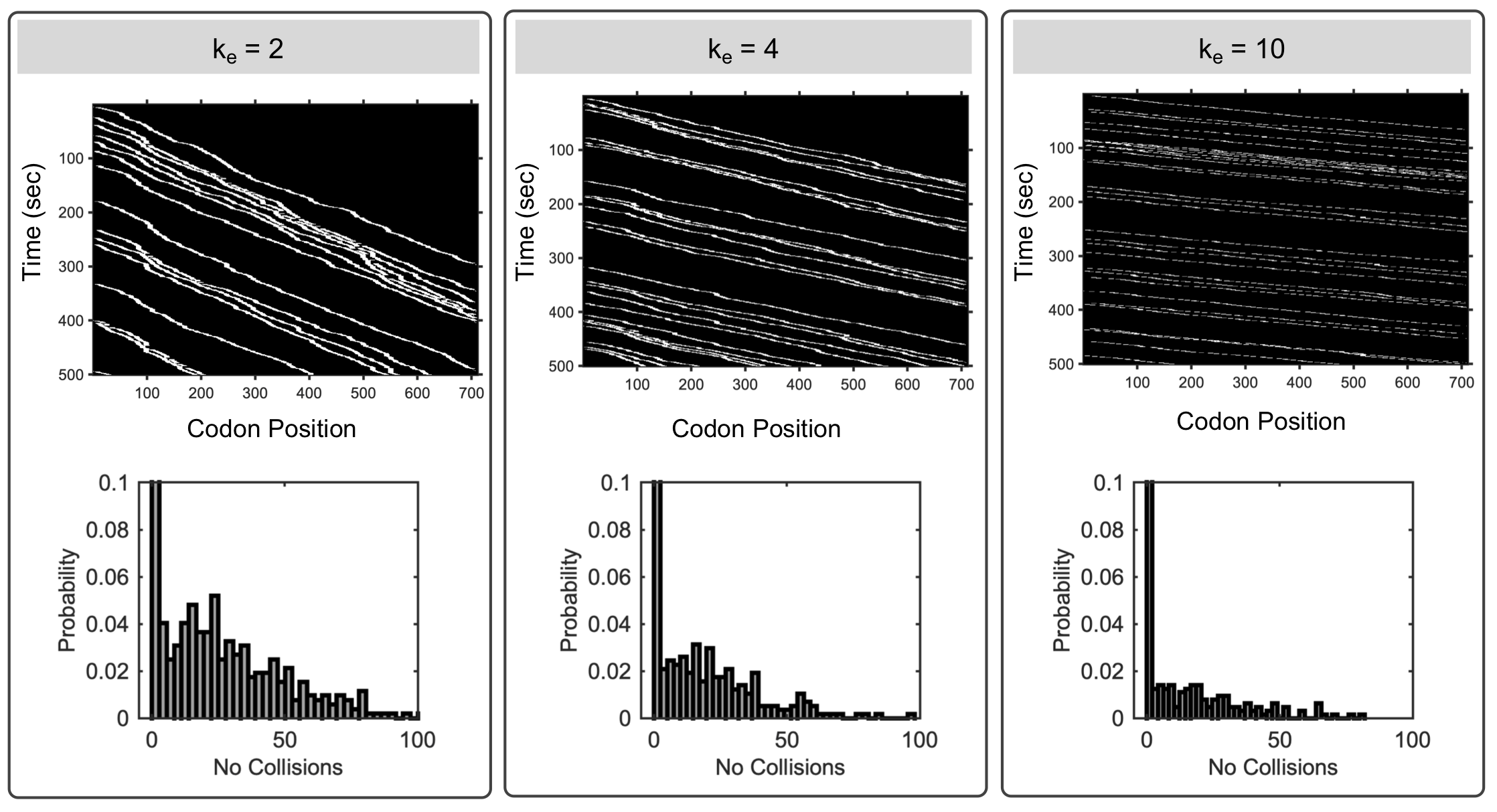

Supplement: S2 Fig — Translation was simulated using the β-actin gene, varying elongation rates, a constant initiation (ki = 0.06 sec−1), and a ribosomal footprint of 9 codons. Top panels show a kymograph of the ribosome movement. Lower panels show the distribution of collisions per each ke. (TIF) [file pcbi.1007425.s002.tif]

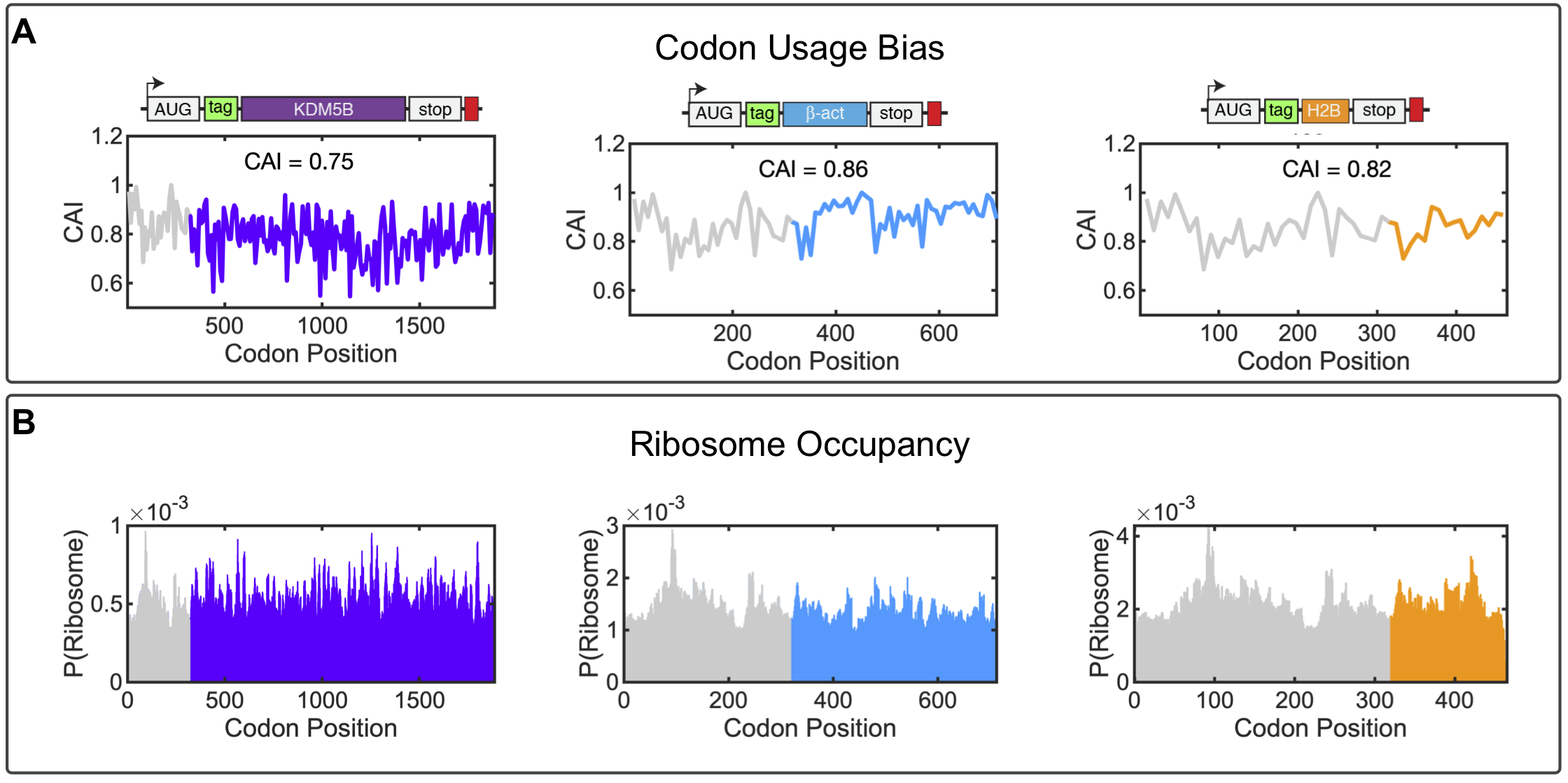

Supplement: S3 Fig — Translation was simulated using the a β-actin gene, varying initiations rates from 0.03 to 0.6, a constant elongation (ke = 10 aa/sec), and a ribosomal footprint of 9 codons. Top panels show a kymograph of the ribosome movement. Lower panels show the distribution of collisions for each ki. (TIF) [file pcbi.1007425.s003.tif]

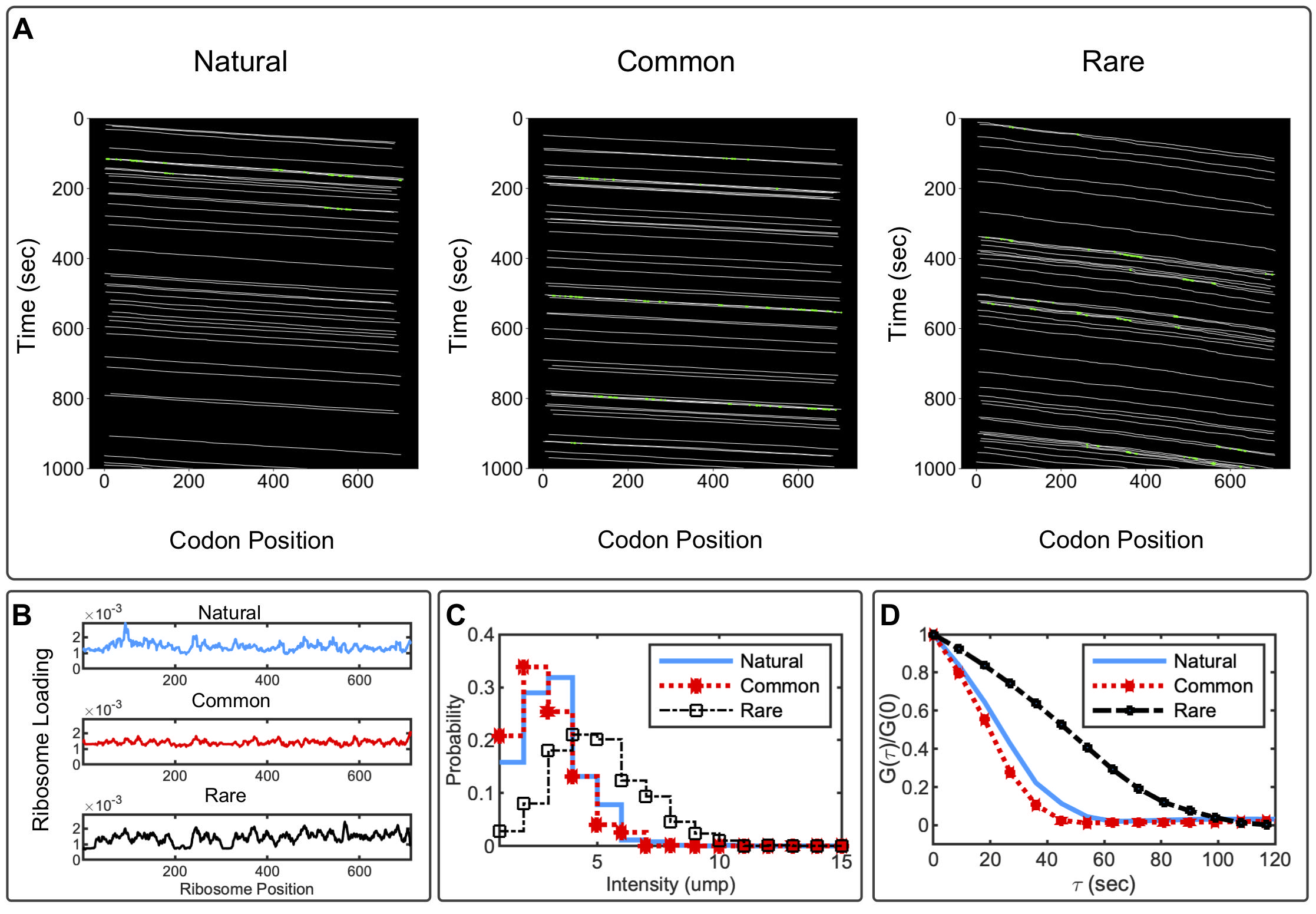

Supplement: S4 Fig — A) Ribosome dynamics for β-actin under different codon optimization constructs (natural sequence, using only common codons, and using only rare codons). In the kymographs, white lines represent the ribosome positions, green spots represent ribosome collisions. The average and standard deviation for the number of collisions is 3.2 ± 0.9 for the natural sequence, 2.4 ± 0.8 collisions for the optimized sequence (common codons), and 6.9 ± 1.5 collisions on the de-optimized sequence (rare codons). B) Ribosome loading for the three codon optimization constructs. D) Auto-covariances calculated for the natural gene sequence, a sequence where all codons are replaced by their most frequent synonymous codon (optimized), and a sequence where all codons are replaced by their less frequent synonymous codon (de-optimized). Simulations were performed using the optimized parameter values given in Eq 29. (TIF) [file pcbi.1007425.s004.tif]

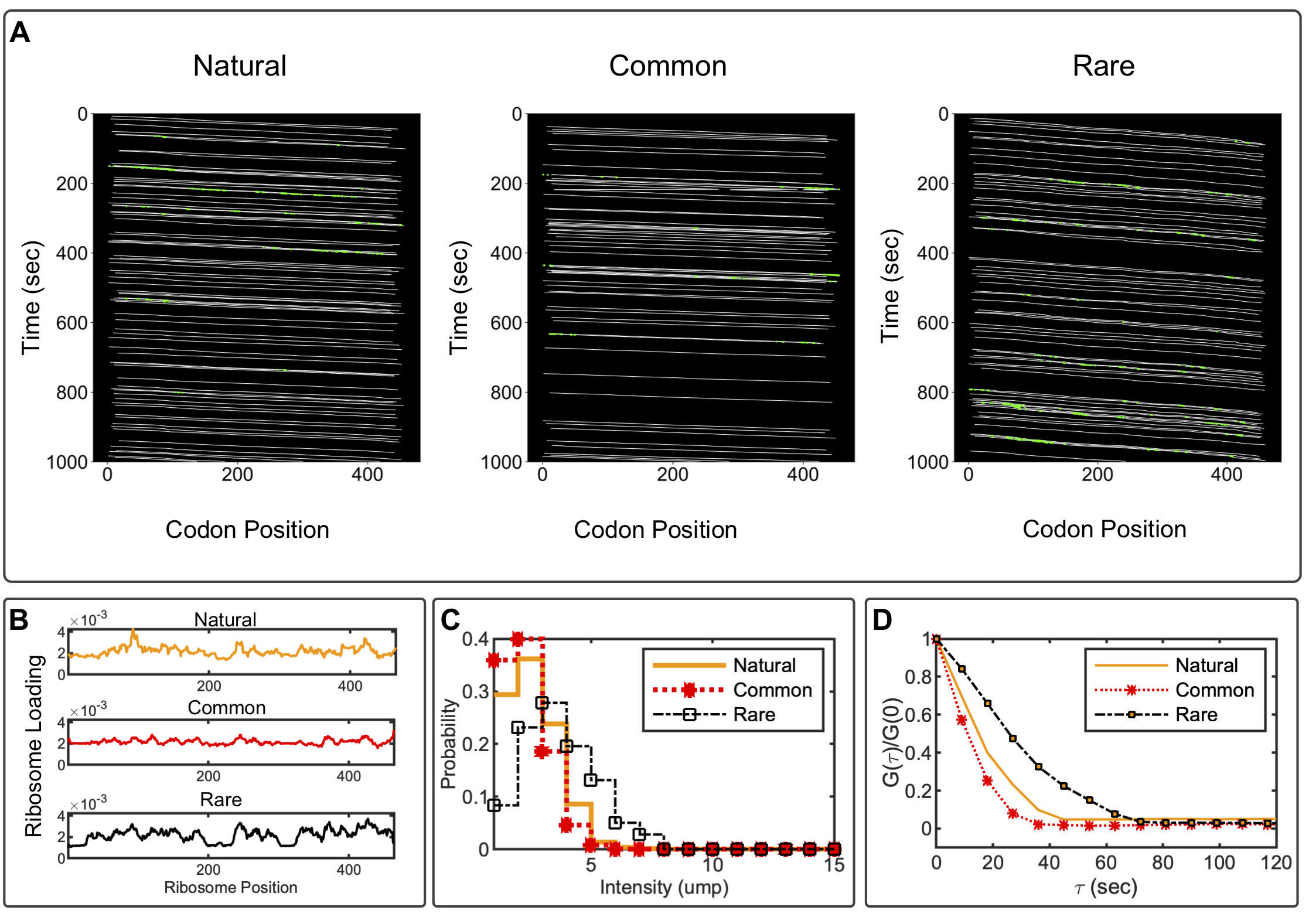

Supplement: S5 Fig — A) Ribosomal dynamics for H2B under different codon optimization constructs (natural sequence, using only common codons, and using only rare codons). In the kymographs, white lines represent the ribosome placement, green spots represent ribosome collisions. The average and standard deviation for the number of collisions is 2.9 ± 0.7 for the natural sequence, 2.0 ± 0.6 collisions for the optimized sequence (common codons), and 6.0 ± 1.1 collisions on the de-optimized sequence (rare codons). B) Ribosome loading for the three codon optimization constructs. D) Auto-covariances calculated for the natural gene sequence, a sequence where all codons are replaced by their most frequent synonymous codon (common), and a sequence where all codons are replaced by their less frequent synonymous codon (rare). Simulations were performed using the optimized parameter values given in Eq 29. (TIF) [file pcbi.1007425.s005.tif]

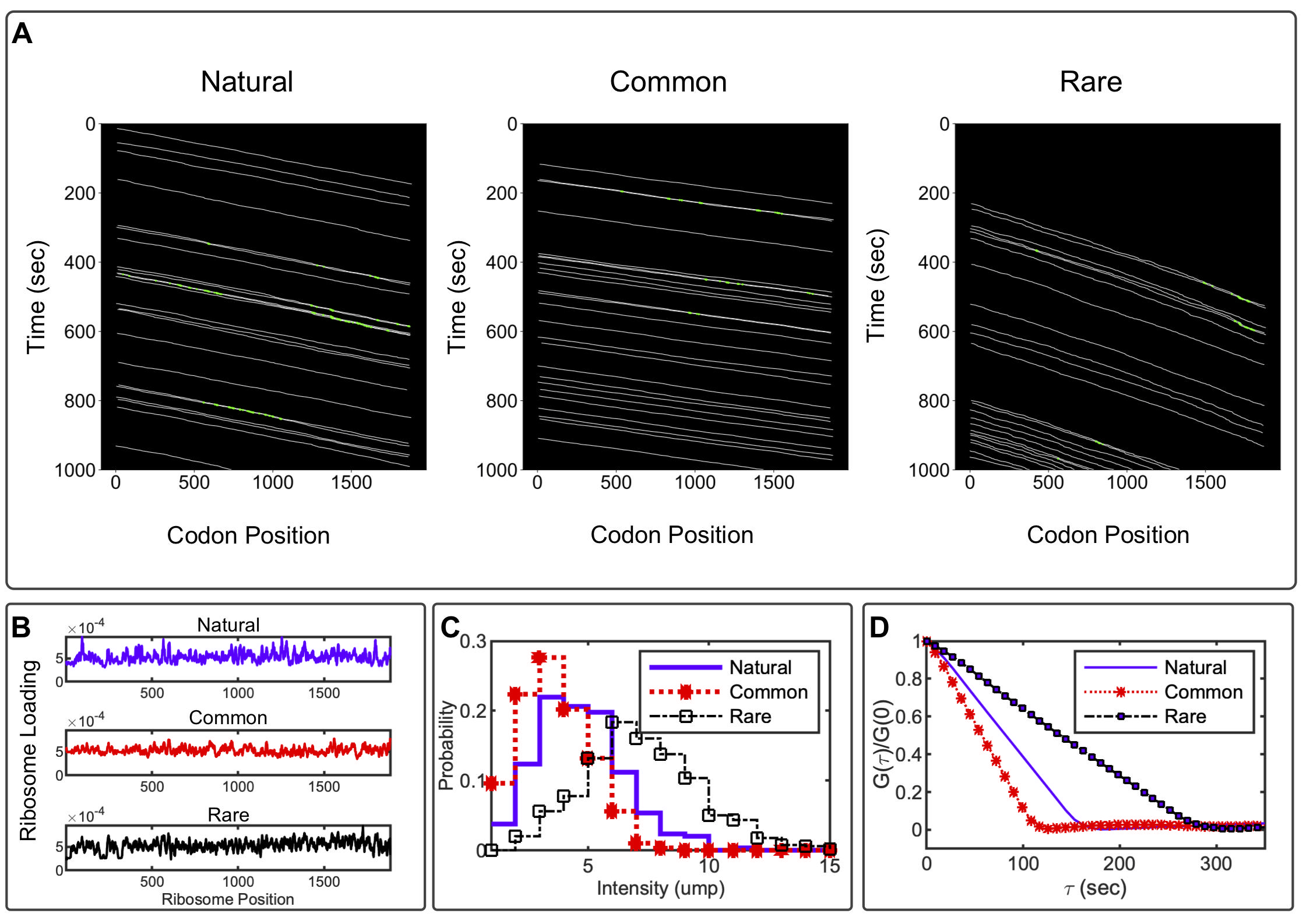

Supplement: S6 Fig — A)Ribosome dynamics for KDM5B under different codon optimization constructs (natural sequence, using only common codons, and using only rare codons). In the kymographs, white lines represent the ribosome placement, green spots represent ribosome collisions. The average and standard deviation for the number of collisions is 4.3 ± 2.0 for the natural sequence, 2.8 ± 1.7 collisions for the optimized sequence (common codons), and 7.8 ± 3.1 collisions on the de-optimized sequence (rare codons). B) Ribosome loading for the three codon optimization constructs. D) Auto-covariances calculated for the natural gene sequence, a sequence where all codons are replaced by their most frequent synonymous codon (common), and a sequence where all codons are replaced by their less frequent synonymous codon (rare). Simulations were performed using the optimized parameter values given in Eq 29. (TIF) [file pcbi.1007425.s006.tif]

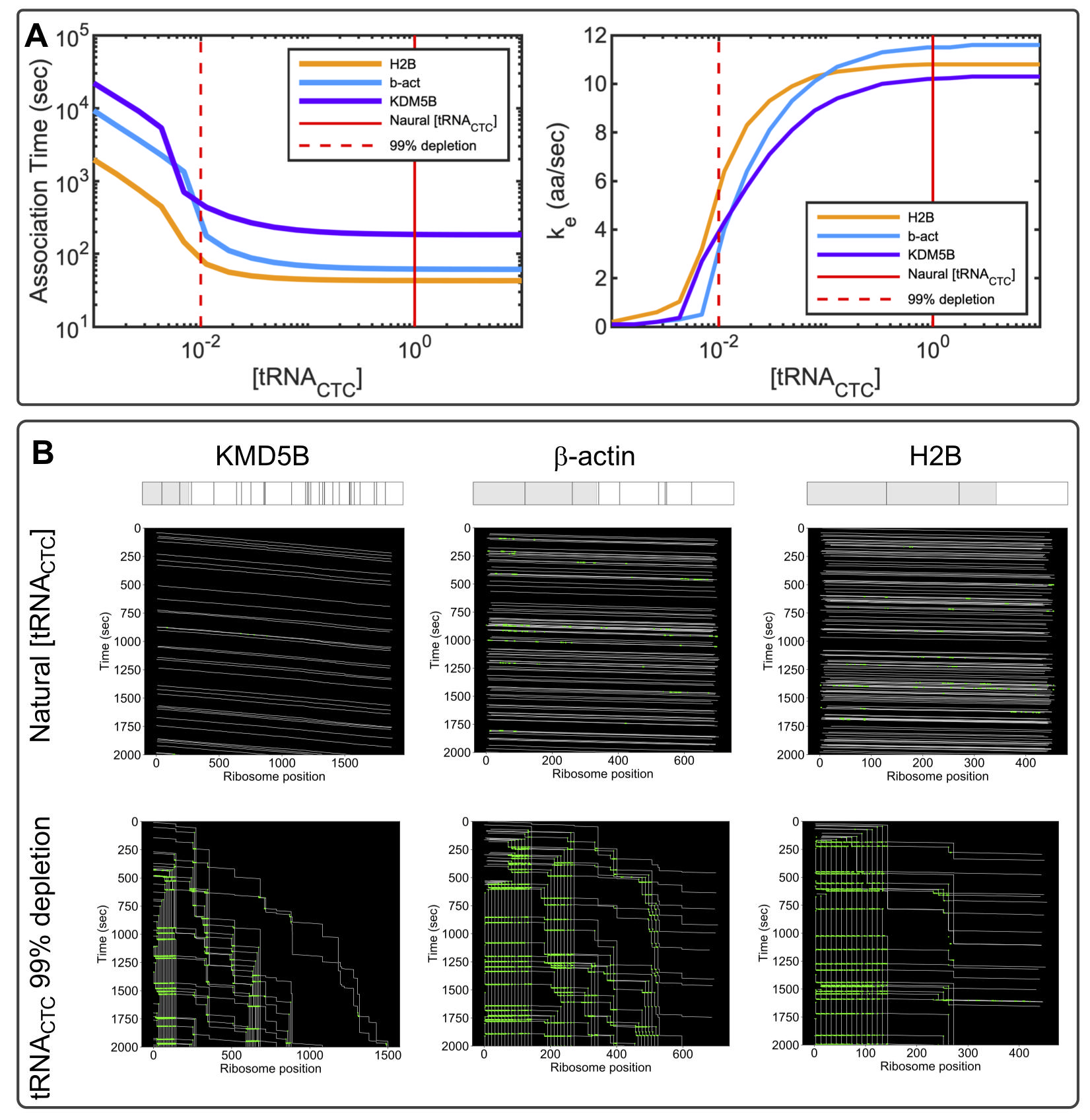

Supplement: S7 Fig — A) Three different genes were studied: KDM5B (magenta), β-actin (cyan) and H2B (orange). Left plot shows the ribosome association time as a function of the tRNACTC concentration. Right plot, shows the calculated elongation rates estimated by dividing the gene length by the average time needed by the ribosome to complete a round of translation. B) Kymographs show the ribosomal dynamics without depletion (upper panels) and with 99% depletion of tRNACTC (lower panels). Above the kymographs, the bar represents the studied gene, and the gray area represents the tag region, black lines denote the positions CTC codons. The frequency of the CTC codon is 29 for KDM5B, 8 for β-actin and 2 for H2B. Simulations were performed using the optimized parameter values given in Eq 29. (TIF) [file pcbi.1007425.s007.tif]

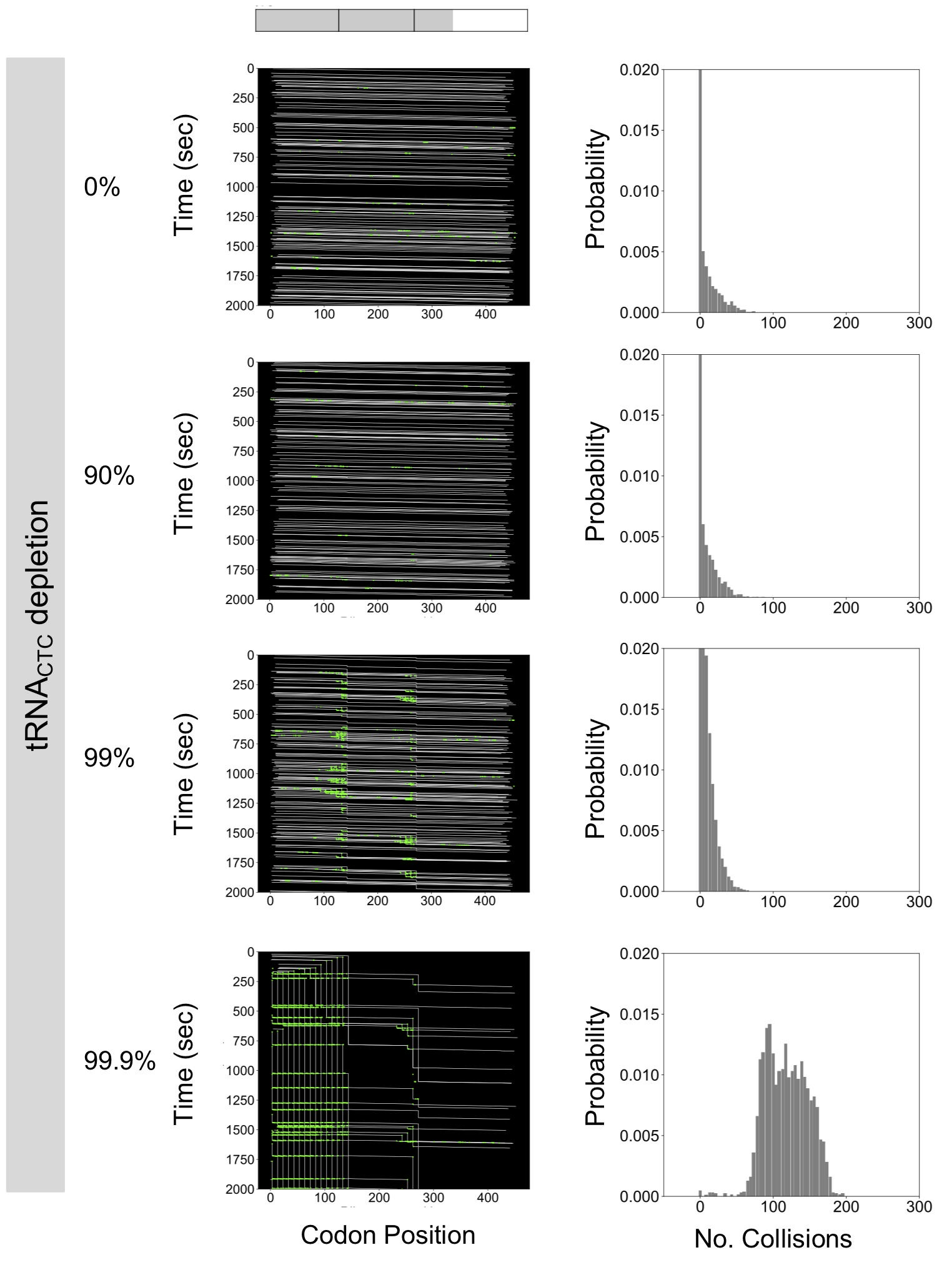

Supplement: S8 Fig — Kymographs (left) show the simulated ribosomal dynamics under different percentages of depletion of tRNACTC. At the top of the kymographs, the bar represents the studied gene, the gray area represents the tag region, and black lines denote the positions of CTC codons. Histograms (right) show the probability of ribosomal collision. Simulations were performed using the optimized parameter values given in Eq 29. (TIF) [file pcbi.1007425.s008.tif]

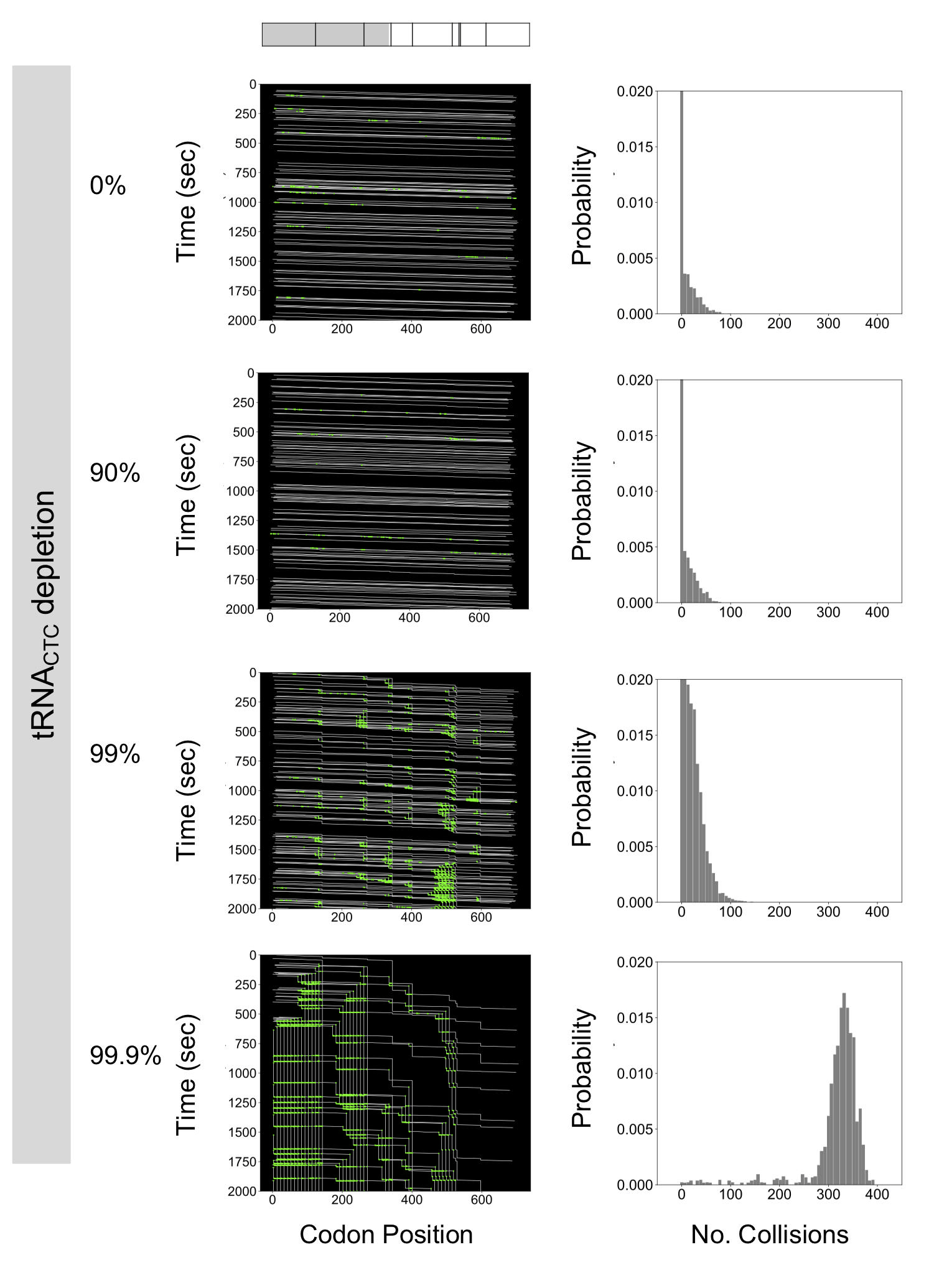

Supplement: S9 Fig — Kymographs (left) show the simulated ribosomal dynamics under different percentages of depletion of tRNACTC. At the top of the kymographs, the bar represents the studied gene, the gray area represents the tag region, and black lines denote the positions of CTC codons. Histograms (right) show the probability of ribosomal collision. Simulations were performed using the optimized parameter values given in Eq 29. (TIF) [file pcbi.1007425.s009.tif]

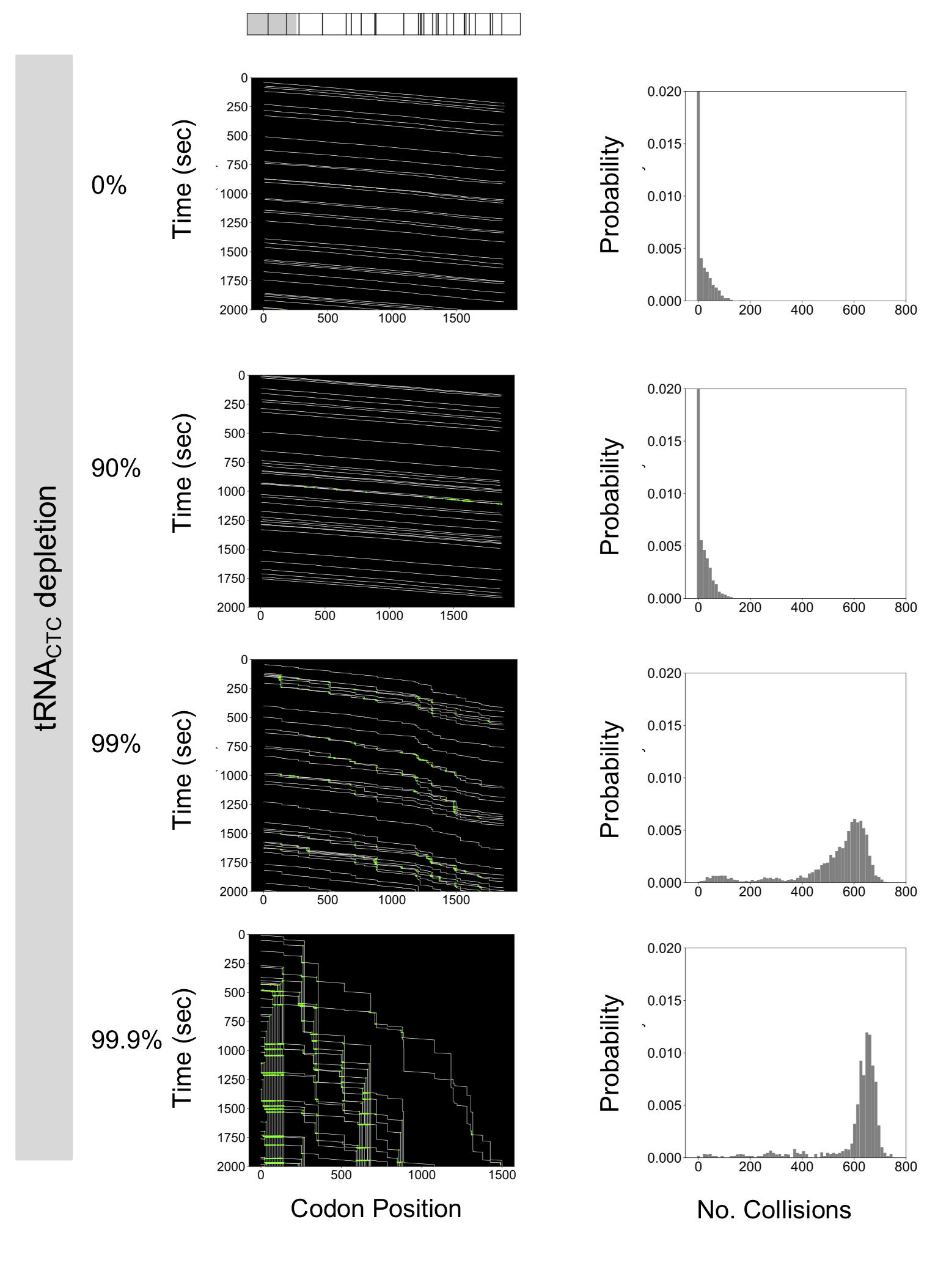

Supplement: S10 Fig — Kymographs (left) show the simulated ribosomal dynamics under different percentages of depletion of tRNACTC. At the top of the kymographs, the bar represents the studied gene, the gray area represents the tag region, and black lines denote the positions of CTC codons. Histograms (right) show the probability of ribosomal collision. Simulations were performed using the optimized parameter values given in Eq 29. (TIF) [file pcbi.1007425.s010.tif]

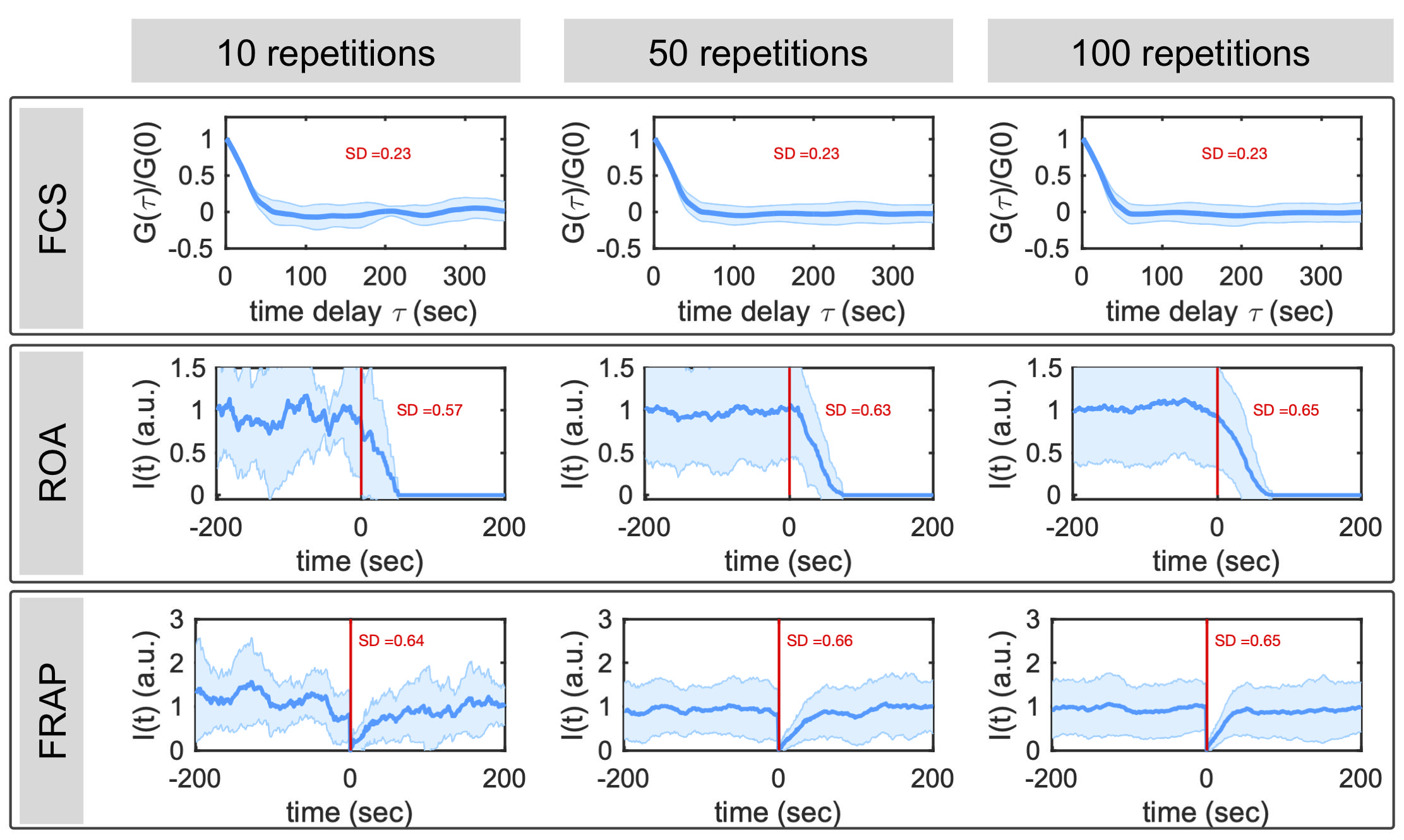

Supplement: S11 Fig — Translation was simulated using the a β-actin gene with the optimized parameter values given in Eq 29. Error bars represent the standard deviation (SD) of the number of repetitions given at the top of each plot. Vertical red lines represent the application of Harringtonine for ROA. Vertical red line represents the time of photobleaching for FRAP. (TIF) [file pcbi.1007425.s011.tif]
