# Supplementary material for: Computational design and interpretation of single-RNA translation experiments
Source: PLoS Comput Biol. 2019 Oct 16;15(10):e1007425. doi: 10.1371/journal.pcbi.1007425 (PMC6816579; doi:10.1371/journal.pcbi.1007425)
Supplement: S1 Table — Codon usage table calculated from the Homo sapiens genome. Table is computed using 93,487 CDS (Coding DNA Sequence), representing a total of 40,662,582 codons [21]. (PDF) [file pcbi.1007425.s012.pdf]

Computational design and interpretation of single-RNA translation experiments.

Luis U. Aguilera, William Raymond, Zachary R. Fox, Michael May, Elliot Djokic, Tatsuya Morisaki, Timothy J. Stasevich, Brian Munsky.

Supplementary Table 1.

Table S1. Codon usage table calculated from the *Homo sapiens* genome. Table is computed using 93,487 CDS (Coding DNA Sequence), that represent a total of 40,662,582 codons, Nakamura, et al., 2000. Reference [21] in main Text.

TTT 17.6	TCT 15.2	TAT 12.2	TGT 10.6
TTC 20.3	TCC 17.7	TAC 15.3	TGC 12.6
TTA 7.7	TCA 12.2	TAA 1.0	TGA 1.6
TTG 12.9	TCG 4.4	TAG 0.8	TGG 13.2
CTT 13.2	CCT 17.5	CAT 10.9	CGT 4.5
CTC 19.6	CCC 19.8	CAC 15.1	CGC 10.4
CTA 7.2	CCA 16.9	CAA 12.3	CGA 6.2
CTG 39.6	CCG 6.9	CAG 34.2	CGG 11.4
ATT 16.0	ACT 13.1	AAT 17.0	AGT 12.1
ATC 20.8	ACC 18.9	AAC 19.1	AGC 19.5
ATA 7.5	ACA 15.1	AAA 24.4	AGA 12.2
ATG 22.0	ACG 6.1	AAG 31.9	AGG 12.0
GTT 11.0	GCT 18.4	GAT 21.8	GGT 10.8
GTC 14.5	GCC 27.7	GAC 25.1	GGC 22.2
GTA 7.1	GCA 15.8	GAA 29.0	GGA 16.5
GTG 28.1	GCG 7.4	GAG 39.6	GGG 16.5
