# Supplementary material for: Computational design and interpretation of single-RNA translation experiments
Source: PLoS Comput Biol. 2019 Oct 16;15(10):e1007425. doi: 10.1371/journal.pcbi.1007425 (PMC6816579; doi:10.1371/journal.pcbi.1007425)
Supplement: Attachment — Submitted filename: Aguilera_et_al_PLOS_CB_2019_Response_Letter.pdf [file pcbi.1007425.s013.pdf]

Oleg A Igoshin
Associate Editor
PLOS Computational Biology

William Noble
Deputy Editor
PLOS Computational Biology

Dear Editors of *PLoS Computational Biology*,

Thank you for your consideration of our manuscript “Computational design and interpretation of single-RNA translation experiments (PCOMPBIOL-D-19-01011, by Luis U. Aguilera, William Raymond, Zachary Fox, Michael May, Elliot Djokic, Tatsuya Morisaki, Timothy J. Stasevich, and Brian Munsky) which we submitted for publication at *PLoS Computational Biology*.

We have revised the manuscript to address all comments from the referees, and we believe that our manuscript and associated computational software has been substantially enhanced.

We want to thank the reviewers for their suggestions, which greatly helped us to improve our manuscript and associated software package. We have carefully taken all of their comments into consideration in preparing our revision, and these have resulted in a manuscript that is more clear and more complete. In the following pages, we provide point-by-point responses to all of the referee comments.

Please do not hesitate to contact us if we can answer any additional questions about our manuscript.

Kind regards,
Dr. Brian Munsky.
Dr. Luis Aguilera

Reviewer's Responses to Questions

Comments to the Authors:

Reviewer #1:

In this paper, the authors introduce a sequence-based stochastic model for simulating detailed translation process. Using this model, the authors generate synthetic data to evaluate three types of single-molecule translation experiment (FCS, ROA, and FRAP) and find that FCS is optimal for estimating translation kinetics. By fitting the model to experimental FCS data for three human genes (KDM5B, β-actin, and H2B), the authors capture the nascent protein statistics and temporal dynamics, and characterize how ribosome activities and translation dynamics vary with different kinetics parameters. To facilitate the application of the model, the authors develop an open-source software package, RNA Sequence to NAscent Protein Simulator (rSNAPsim), which allows simulating the single-molecule translation dynamics of any gene.

I found the manuscript very suitable for PCB, in terms of both the subject matter and the methodology. There are only a few small points that I'd like the authors to address before publication:

(1) In the title of the paper, it is not clear what "computational design" means. The term is not used or defined in the rest of the paper. Do the authors mean that they have applied the computational approach to compare three different experimental assays of measuring translation dynamics? Please clarify in the main text or change the title.

We fully appreciate this observation. To clarify the use of "Computational design" from the title of this manuscript, we define our use the words "computational design" in the introduction and discussion as follows:

Line 69 (new document):

"As these experimental techniques rapidly evolve, they induce a growing need for precise and flexible computational tools to interpret the resulting data and to design the next wave of single-RNA translation experiments. To help fill this gap, we present a versatile new set of computational design tools to estimate which specific single-mRNA translation dynamics experiments would provide the most accurate inference of model parameters."

Line 619 (new document) in the discussion:

"On the one hand, our model can help to explain these differences (S11_Fig), but more importantly, the models themselves can be used to simulate and evaluate different computational designs to determine which are more likely to reveal important biophysical mechanisms or parameters. We envision that user-friendly simulations, such as those provided by rSNAPsim, can be used to optimize combinations of probe placement, gene length, codon usage differences, video frame rates, drug-based perturbations, or specifications of movie length."

(2) Equation 1 of the paper has several problems: (a) the term “w_i(x_2,…,x_nf+i)” should be “w_1(x_1,…,x_nf+1)”; (b) the term “w_i+1(x_i+1,…,x_nf+i+1)” should be “w_i(x_i,…,x_nf+i)”, (c) there should be ellipsis on the left side of “x_i”.

Thank you for catching these typographical errors. These have been corrected.

(3) There are multiple language issues in the manuscript. Following is a list of them. The authors and the editorial team will have to make sure all such errors are corrected.

Thank you for helping us to detect misspelled sentences. We carefully checked the grammar and spelling in this revision, and fully copyedited the document.

• In the abstract of the paper, “Finding that FCS analyses are optimal for short or long length genes” should be “Finding that FCS analyses are optimal for both short and long length genes”.

Corrected.

• In the abstract of the paper, “we introduce a new open-source software package, … (rSNAPsim) to easily simulate …” should be “we introduce a new open-source software package, … (rSNAPsim), to easily simulate …”.

Corrected.

• In the abstract of the paper, “…
to easily simulate the single-molecule dynamics of any gene sequence …” should be “…
to easily simulate the single-molecule translation dynamics of any gene sequence …”.

Corrected.

• In line 2 of the paper, “fluorescence time lapse microscopy” should be “time-lapse fluorescence microscopy”.

Corrected.

• In line 19 of the paper, “Yet, despite their overwhelming importance …” should be “Despite their overwhelming importance …”.

Corrected.

• In line 23 of the paper, “Imaging single-molecule transcription was first achieved …” should be “Single-molecule imaging of transcription was first achieved …”.

Corrected.

• In line 43 of the paper, “For example, Morisaki and Stasevich, [12] recently reviewed …” should be “For example, Morisaki and Stasevich recently reviewed … [12]”.

Corrected.

• In line 52 of the paper, “run off assay” should be “run-off assay”.

Corrected.

• In lines 80-81 of the paper, “single-molecule dynamics of any gene” should be “single-molecule translation dynamics of any gene”.

Corrected.

• In lines 145 of the paper, “Nascent Transcript Kinetics” should be “Nascent Translation Kinetics”.

Corrected.

• In lines 281-282 of the paper, “we obtained RMSE_ROA > 2.0 sec_-1 …, Fig 3B” should be “we obtained RMSE_ROA > 2.0 sec_-1 … (Fig 3B)”.

Corrected.

• In line 354 of the paper, the term “effects of parameters” is confusing. Do the authors mean “exploring how ribosome activities and translation dynamics vary with different parameters”? Please consider modifying the subtitle.

We modified the subtitle in line 396 (new document) to :
“Exploring How Translation Dynamics Vary With Different Parameters”

• In line 400 of the paper, the term “lower fluorescence intensity distributions” is confusing, since the intensity distribution is a function, not a single value. Please clarify what property of the distribution is lower.

Thanks for this observation. We simply refer to the overall value of the fluorescence intensity. Following the reviewer’s suggestions, we corrected the paragraph in line 438 (new document) to:

“In all cases, optimized gene sequences speed-up ribosome dynamics, and de-optimized sequences cause a slower elongation rate that is observed in the auto-covariance plots given in S4_FigD, S5_FigD, and S6_FigD. Moreover, for constant initiation rates, faster elongation would lead to lower ribosome loading (S4_FigB, S5_FigB, S6_FigB) and therefore lower fluorescence intensities, as shown in the distributions given in S4_FigC, S5_FigC, and S6_FigC.”

• At the end of line 515, the authors should start a new paragraph for the discussion of the FRAP experiment.

Corrected.

• In line 541 of the paper, “the the corresponding tRNA” should be “the corresponding tRNA”.

Corrected.

• In lines 561-562 of the paper, “all of the computational analyses describe above” should be “all of the computational analyses described above”.

Corrected.

• In line 563 of the paper, the term “multi-frame and multi-color translation” is confusing. Please clarify what it really means.

We agree with the reviewer, the term “multi-frame and multi-color” was not well described in the previous manuscript. By ‘multi-frame’ translation, we refer to the frameshifting dynamics recently described by Lyon et al [14] and by ‘multi-color’ we refer to the new multi-color fluorescence system developed in [14] and [15]. We changed this paragraph to:

“Furthermore, all of the computational analyses described above are easily adapted to allow for analysis of simultaneous multi-frame translation dynamics (e.g., when translation occurs on overlapping open reading frames as is the case during frame-shifted translation), as we implemented and described in [14]. Similarly, the code is easily extended to analyze translation of genes that contain more than one set of fluorescence tags in multiple colors, as has been explored experimentally in [15].”

Reviewer #2:

In this manuscript by Aguilera et al., the authors implement a method for analyzing single mRNA translation data using the SunTag/MS2 system. The approach is based on time-lapse imaging of fluorescence which corresponds to the synthesis of nascent protein. Experimentally, the data can be acquired for fluctuation analysis, fluorescence recovery after photobleaching, and run off, all of which are treated in the manuscript. Computationally, the data can be understood using a master equation approach which the authors then solve in a few limiting cases. Finally, they implement an analysis package based on stochastic simulations. This paper does not report any new biological findings per se, and the theoretical advance is minimal if any. They test their computational model on thousands of mRNA in silico and reach the conclusion that fluctuation analysis is the most versatile and accurate analysis tool. In summary, analyzing this type data is certainly nuanced and difficult, and there are already a number of experimental labs working in this area. Providing practitioners a tool to analyze fluorescence trajectories, be they from translation or transcription, solves a problem and makes a novel contribution.

Major Comments:

1. The model for single-molecule translation is the same as the model for single-molecule transcription published recently (PMID: 30554876). While I appreciate that the authors have tailored it to translation and parameterized it for codon usage, the math is the same as that presented in the supplement of that paper. At a minimum, the authors should indicate which equations are the same and which are different.

We deeply thank the reviewer for sharing this with us. We were not aware of the model presented in the supplemental data of Rodriguez et al., earlier this year. We have revised our manuscript first to acknowledge that existing article, both when we introduce our model (Lines 85 and 86) and later in the discussion (Line 506).

We have also revised our manuscript to highlight the extensions that our efforts provide beyond that analysis. The main differences between previous transcription models and our model are as follows:

- Our translation models (including the full stochastic simulations and our reduced theoretical expressions) are analyzed at single-codon resolution. Although Rodriguez's Master Equation model was formulated at single-nucleotide resolution, it was coarse-grained prior to its solution (the final model in Rodrigues et al. consisted of 2 gene states and only 3 RNA elongation steps).
- We introduce variability of translation with codon-dependent rates. While such mechanisms may not make as much sense for analysis of transcription, they can be highly relevant in translation, as we shown in our analyses of common/natural/rare codon usage (Fig S4 to S6) and of tRNA depletion (Fig. S7).
- We analyze the overall ribosome association time, loading statistics (mean and variance), and auto-covariance function using this single-codon-resolution and codon-dependent model. The Master Equation model specified in Rodriguez et al., could in

principle be used to compute the ribosome loading statistics, but that model was not extended to the computation of auto-covariances.
- We consider an adjustable ribosome exclusion volume (20 to 30 nucleotides or 9 to 10 codons), compared to the nearest neighbor exclusion considered in Rodriguez et al.
- Our introduction of the probe intensity vector (Eqn. 5) allows for analyses of arbitrary numbers and positions of tags in the gene construct, which has a real added value to the final user. Some example applications are:
    - To simulate and compare the intensity fluctuations of two gene constructs, with different numbers of tags.
    - To compare the effects to put the tag region in different locations, for example, N- versus C- terminus of the elongating protein.

To address these issues in our previous manuscript, we have updated the text of our manuscript as follows:

Line 84
“To simulate translation with single-molecule resolution, we adopted a stochastic model of polymerization that is similar to those developed previously in references [Gorgoni2016, Bonnin2017, Rodriguez2019]. We then extend this model to allow for variable ribosome sizes, codon- and tRNA-dependent translation elongation rates, and arbitrary placement of fluorescent probe binding epitopes, and we analyze these models specifically in the context of single-mRNA translation as observed using time-lapse fluorescence microscopy experiments (e.g., Fig. 1).”

Lines 501 to 513
“Imaging translation in living cells at single-molecule resolution is a new experimental technology that has been applied to only a few genes so far [Morisaki2016, Wang2016, Wu2016, Pichon2016, Yan2016, Lyon2019, Boersma2019], but the number of such studies is expected to grow considerably in the near future [Morisaki2018]. Computational models can aid in this research by extracting improved biophysical understanding and parameters from single-molecule data. For example, in related analyses of transcription dynamics, Rodriguez et al., [Rodriguez2019] used a coarse grained stochastic model to capture the polymerase elongation process and reproduce transcription dynamics for a multi-state promoter. Here, we extended that theoretical framework to include the most widely accepted mechanisms affecting nascent protein translation, including codon-dependent elongation and ribosome interference [Bonnin2017] and with specific attention to the placement of fluorescent probes. To complement previous models that have sought to reproduce data from earlier bulk cellular assays [Gorgoni2016], and ribosome profiling data [Gritsenko2015,Duc2018], our focus has been to integrate single-mRNA stochastic dynamics models with data from in vivo single-RNA translation dynamics experiments.”

2. There is real added value in comparing the different techniques for quantifying fluorescence time traces. One thing that wasn’t clear to me: is all the fitting done with the simulated Gillespie approach? It should be. In this era, there is really no point in using approximations when there are so many fast simulators. I imagine that is what is implied by this sentence: “this simulator

performs stochastic simulations considering the widely accepted mechanisms ribosome elongation, such as codon usage and ribosome interference."

We thank the referee for raising this point, as it has helped us to clarify our intentions in this manuscript.

Indeed, as the referee had expected, all of our analyses of real experimental data use the full non-linear stochastic simulations, which we have optimized to be exceedingly fast.

However, we believe that there is substantial value in the development of simple (yet validated) theoretical analyses for two related objectives:

First, reduced theoretical models allow us to gain a deeper understanding of the system dynamics. Equations 21 to 23 (numbering in new manuscript) give simple estimates for the sensitivity of observable traits (e.g., mean and variance of ribosome loading or characteristic elongation times) to mechanistic parameters (e.g., initiation rates or elongation rates for specific codons).

Second, simple algebraic estimates like those in Equations 21 - 23 are orders of magnitude faster to compute than are the results of simulations. Such efficiency is paramount when one seeks to perform parameter estimation for vast combinations of different genes (e.g., our considered library of 2,647 genes) and in different experiment designs (e.g., our consideration of 24 different combinations experiments, different frame rates, and different numbers of spots) as we accomplished in Fig 3.

(Relocated from Referee Point 1) In a related point, some of the early reading is a bit tedious and could be condensed or included in a supplement. For example, the theory section builds to two equations (23, 24) which are quite similar to those reported in Ref. 2.

As we replied above, we believe there is merit in simple theoretical analyses that are much more efficient than simulations. But, before one can confidently use simplified analyses, it is necessary to demonstrate the validity of these simplifications. That verification was the main objective of the first part of our results section.

However, we understand the concerns of the reviewer regarding flow of the paper. To address her/his comments, we have moved the model development to a new section prior to our 'Results" section, and we have added text to provide improved motivation and justification for our model simplifications as follows:

Line 154:

"Simplifications for combinatorial analyses of genes, parameters, and experiment designs.

The model as defined above is sufficient to simulate fluorescence dynamics for any specified gene and for a vast range of potential time lapse microscopy experiments. However, these simulations become computationally intensive when studying combinations of thousands of genes, using thousands of different parameters sets, and for hundreds of different experiment designs. To ameliorate this concern, we next introduce model simplifications that progressively remove elements from the original model, such as ribosome exclusion and single-codon resolution, while retaining effects of codon-dependent translation rates and the geometric placement of fluorescent tags. We then test under what conditions (i.e., parameters and gene lengths) these simplifications are valid, and we compare these conditions to experimentally reported values."

3. Why is the autocorrelation always normalized by G(0)? This quantity contains the information about initiation rate. In a related point, why does the raw fluorescence trace have to be normalized by the individual intensity? The fluctuations should be sufficient to back out the occupancy. To be clear, what biophysicists call 'autocorrelation' is actually the 'autocovariance' and usually contains an un-normalized amplitude.

We thank the referee for pointing out improper use of 'auto-correlation', and we have adjusted our manuscript to use 'auto-covariance' or 'auto-covariance coefficients' when we are discussing the normalized auto-covariance. We believe that this is more precise.

We agree with the reviewer that auto-covariance coefficients neglect information concerning the ribosome loading variance, and specifically the initiation rate. Indeed, we performed this normalization specifically to isolate our experimental evidence concerning ribosome loading distributions from that concerning fluorescence fluctuation time scales. To quantify ribosome loading distributions, we collected direct measurements of the fluorescence intensity distributions using large numbers (>100) independent spots, but at higher laser powers and for much shorter trajectories. These intensities were calibrated directly against a second construct with a single FLAG epitope at high enough laser powers to permit single-fluorophore detection. Through this analysis, we obtained more precise estimates of intensity distributions, and we fit our models to these full intensity distributions (e.g., Fig 4 middle plots). Unfortunately, at these high laser powers, it is not possible to quantify long trajectories due to excessive photo-bleaching and photo-toxicity. At lower laser powers, we could perform single-mRNA tracking and obtain estimates of the auto-covariance, but the signal-to-noise is worse, and the variance of fluorescence intensity becomes dominated by shot noise. Therefore, to make simultaneous use of high laser power distribution data and low laser power fluctuation data, we normalized out the variance from the auto-covariance plots.

We have updated the manuscript to be more clear on this choice and its motivation. Moreover, we also note that in our simulations, we normalize auto-covariances by the simulated variance, G(0), which we can compute directly. For the experimental data, we cannot measure G(0) directly because it is dominated by shot noise, so we instead interpolate G(0) using a linear extrapolation from the first four points of the measured auto-covariance function. This new normalization resulted in improved fits as shown in Figure 4, with new estimated parameters, line 395 and Eq.

31. To maintain consistency in our analyses, we repeated the experiments for the right patel in figure 3, obtaining almost identical results as the previous version of the paper, lines 291 to 294. Finally, with simply updated figures S4 to S11 to consider the new optimized parameters values.

4. Averaging experimental autocorrelations is not trivial and was discussed in Ref. 35. Specifically, for image-based analysis (as done here), the traces are often short and of variable length and may contain few translation events. The sparseness of the data represents a problem for fluctuation analysis. As such, the arithmetic average is not strictly correct. If the goal is to generate a broadly useful analytic tool, I strongly recommend implementing an averaging scheme which is specific to the translation data in the distributed program.

We fully appreciate the observation concerning the normalization of the autocorrelation. We have updated all analyses in our GUI implementation to provide the user option to implement each of the normalization approaches described in Ref 30 (Coulon and Larson 2016, previously Ref. 35). Specifically, the user can calculate the auto-covariances using the intensity mean (Equation 6 in Reference 30), or the global intensity mean (Equation 7 in Reference 35). We have also included our experimental data as a test set in our GUI toolkit, so that it is clear to the user how these choices affect the presentation of the experimental data. Screenshots of this comparison are shown below in Figures 1 A and B.

Figure 1A. GUI screenshot. Calculating the autocorrelation function by normalizing the intensity by the mean intensity of each trace.	Figure 2B. GUI screenshot. Calculating the autocorrelation function by normalizing the intensity by global mean intensity.
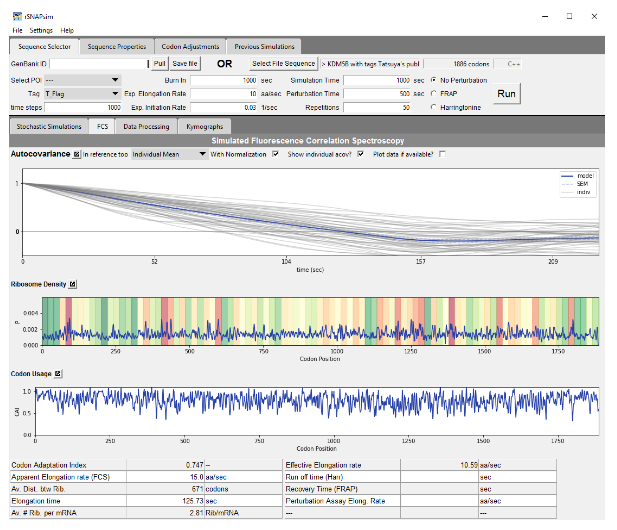	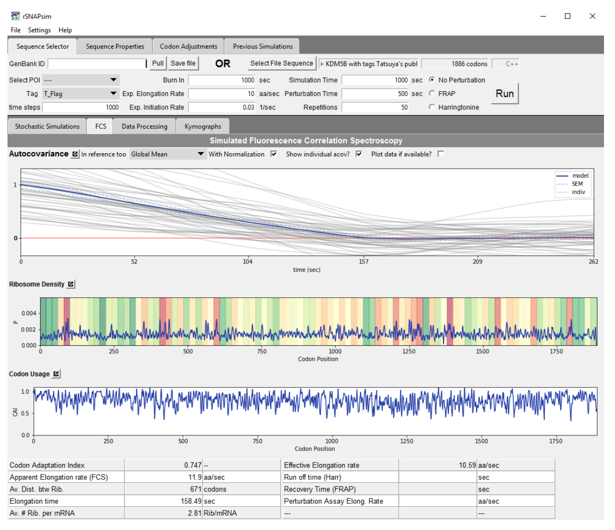

For our particular data sets, we found that use of the global mean would problematic due to substantial variability in the mean intensities for different spots. Because laser powers were tuned to the lowest possible settings to allow tracking of long trajectories, we encountered substantial variations in levels of illumination for spots contained in different cells (e.g., with different shapes and sizes), different locations within cells, or from imaging cells on different days. Despite dissimilarities in the means and variances of intensity levels among different trajectories, the

fluctuation timescales for each gene were well-conserved from spot-to-spot for a given mRNA time yet distinct from one mRNA type to another.

In addition to these changes, we include a more rigorous approach to compare the model and data. Specifically, we included the likelihood functions given in Eqs. 28 and 29, to compare auto-covariances, similar analyses are also used in Ref 30 (Coulon and Larson 2016, previously Ref. 35). For intensity distributions we used the likelihood function given by Eqs. 26 and 27.

5. How is translational bursting accommodated in the model?

We have not specifically incorporated bursty kinetics into the model as described here because bursty translation is not as well understood for translation kinetics as it is for transcription kinetics, and bursting behavior is not necessary to reproduce any of the data contained in this manuscript. However, bursty dynamics can be incorporated easily into our full models as well as into our simplified analysis of auto-covariances. This can be accomplished by adding two additional mRNA states (for the simplest case) corresponding to 'ON' or 'OFF' mRNA species and two reactions to represent activation and inactivation of the translation initiation mechanisms. This would require a couple straightforward changes to Eqns. 2 (add linear dependence on the ON state) and Eqns. 6 and 8 (two new rows and columns in S, W_0 and W_1 corresponding to new species and new reactions). Everything else in our analyses would be unaffected. We speculate that bursty dynamics are an important mechanism taking place during translation initiation, but at this point, the parameters for our reported genes are still unknown. Therefore, in the absence of specific experimental need to include bursting kinetics at this time, and in the interest of simplicity, we have opted not to include bursting kinetics in the current manuscript.

Have all data underlying the figures and results presented in the manuscript been provided?

Reviewer #1: Yes

Reviewer #2: No: image data is not provided
All data and source code used in this project are available in the following repositories.
https://github.com/MunskyGroup/Aguilera_PLoS_CompBio_2019.git
https://github.com/MunskyGroup/rSNAPsim.git
